# Acute stress imparts a transient benefit to task-switching that is not modulated following a single bout of exercise

**DOI:** 10.3389/fpsyg.2023.1157644

**Published:** 2023-07-18

**Authors:** Anisa Morava, Benjamin Tari, Joshua Ahn, Mustafa Shirzad, Matthew Heath, Harry Prapavessis

**Affiliations:** ^1^Faculty of Health Sciences, School of Kinesiology, University of Western Ontario, London, ON, Canada; ^2^Department of Psychiatry, University of Oxford, Oxford, United Kingdom; ^3^Canadian Centre for Activity and Aging, University of Western Ontario, London, ON, Canada; ^4^Graduate Program in Neuroscience, University of Western Ontario, London, ON, Canada

**Keywords:** antisaccades, cognitive flexibility, executive function, oculomotor, psychosocial stressor

## Abstract

**Introduction:**

Cognitive flexibility represents a core component of executive function that promotes the ability to efficiently alternate—or “switch”—between different tasks. Literature suggests that acute stress negatively impacts cognitive flexibility, whereas a single bout of aerobic exercise supports a postexercise improvement in cognitive flexibility. Here, we examined whether a single bout of aerobic exercise attenuates a stress-induced decrement in task-switching.

**Materials and Methods:**

Forty participants (age range = 19–30) completed the Trier Social Stress Test (TSST) and were randomized into separate Exercise or Rest groups entailing 20-min sessions of heavy intensity exercise (80% of heart rate maximum via cycle ergometer) or rest, respectively. Stress induction was confirmed via state anxiety and heart rate. Task-switching was assessed prior to the TSST (i.e., pre-TSST), following the TSST (i.e., post-TSST), and following Exercise and Rest interventions (i.e., post-intervention) via pro- (i.e., saccade to veridical target location) and antisaccades (i.e., saccade mirror-symmetrical to target location) arranged in an AABB task-switching paradigm. The underlying principle of the AABB paradigm suggests that when prosaccades are preceded by antisaccades (i.e., task-switch trials), the reaction times are longer compared to their task-repeat counterparts (i.e., unidirectional prosaccade switch-cost).

**Results:**

As expected, the pre-TSST assessment yielded a prosaccade switch cost. Notably, post-TSST physiological measures indicated a reliable stress response and at this assessment a null prosaccade switch-cost was observed. In turn, post-intervention assessments revealed a switch-cost independent of Exercise and Rest groups.

**Conclusion:**

Accordingly, the immediate effects of acute stress supported improved task-switching in young adults; however, these benefits were not modulated by a single bout of aerobic exercise.

## Introduction

Executive function represents a constellation of higher-order processes including the core components of inhibitory control, working memory, and cognitive flexibility (Diamond, 2013). Executive function and health are interrelated such that executive function behaves as an antecedent and consequence of health behaviors (for reviews, see Allan et al., 2016; Williams et al., 2017). Indeed, an extensive literature has shown that executive function is modulated via a number of external factors including stress and exercise (Chang et al., 2012; Plieger and Reuter, 2020). In the first case, stress represents a state of threatened homeostasis (Chrousos and Gold, 1992) or an experience wherein an individual appraises a situation as exceeding their resources and endangering their wellbeing (Lazarus and Folkman, 1984). Shields et al.’s (2016) meta-analysis concluded that an acute bout of stress provides a transient disruption to the components of executive function. In particular, the authors reported stress impaired performance on tasks assessing cognitive flexibility and working memory. In contrast, effects of acute stress on inhibitory control were mixed. Importantly, cognitive flexibility requires the efficient ability to inhibit extraneous stimuli and hold a currently relevant task set in mind. The stress-induced reduction in executive performance has been attributed to the reallocation of “finite” executive function resources in conjunction with neurobiological alterations associated with activation of the hypothalamic-pituitary-adrenal (HPA) and sympathetic-adrenal-medullary (SAM) axes (Mather and Sutherland, 2011; Plessow et al., 2011; Shansky and Lipps, 2013). In the second case, meta-analytic evidence suggests single bouts of aerobic and/or resistance exercise provide a brief (i.e., <60-min) benefit to executive function (Lambourne and Tomporowski, 2010; Chang et al., 2012; Ludyga et al., 2016). This effect has been attributed to exercise-based increases in cerebral blood flow (Tari et al., 2020), biomolecule concentrations (Zouhal et al., 2008; Knaepen et al., 2010) and resting state functional connectivity (Schmitt et al., 2019) that support improved efficiency of local neural circuits (Moore and Cao, 2008). Notably, a narrative review by Sandi (2013) highlighted stress response systems such as the HPA and SAM axes are heavily integrated with regions supporting executive functions (Joëls and Baram, 2009). A single bout of exercise has also been demonstrated to buffer the effects of stress and promote stress-related recovery as determined by biological (e.g., heart rate, blood pressure, cortisol levels) and perceptual (e.g., State Trait Anxiety Inventory) measures of stress (LaManca et al., 2001; Brownley et al., 2003; Alderman et al., 2007; Wunsch et al., 2019). Taken together, this underscores the use of a single bout of exercise in attenuating the deleterious effects of acute stress.

To our knowledge, however, no research has directly examined the potential by which a single bout of exercise may blunt a stress-induced decrement in executive function. In particular, the current work explored whether a 20-min heavy intensity (80% HR maximum) aerobic exercise bout via cycle ergometer attenuates stress-induced impairments in the cognitive flexibility component of executive function via task-switching. For example, a number of studies by our group have examined cognitive flexibility via a paradigm wherein individuals alternate between pro- and anti-saccades in predictable (i.e., AABB) or unpredictable (i.e., AABABB…) task-switching schedules (Weiler and Heath, 2012a,b, 2014a; Heath et al., 2015; Weiler et al., 2015). Prosaccades are a goal-directed eye movement (i.e., saccade) directed to a veridical target location, whereas antisaccades entail a response mirror-symmetrical to a target. Antisaccades produce longer reaction times (RTs; Hallett, 1978) and “less accurate and more variable endpoints than prosaccades (Gillen and Heath, 2014) and these behavioral ‘costs’ have been linked to the top-down executive processes of response suppression (i.e., inhibiting a reflexive prosaccade) and vector inversion (i.e., 180° spatial transformation)” (for review see Munoz and Everling, 2004). Further, when a prosaccade is preceded by an antisaccade (i.e., task-switch trial) RTs are “longer than when preceded by its same task-type (i.e., task-repeat trial), whereas antisaccade RTs for task-switch and task-repeat do not reliably differ” (Weiler and Heath, 2012a,b). The basis for the asymmetrical switch-cost is that the executive demands of antisaccades result in a task-set inertia that proactively delays the planning of a subsequent prosaccade (i.e., the unidirectional switch-cost) (Weiler and Heath, 2012a). Notably, Shukla and Heath (Shukla et al., 2020; Shukla and Heath, 2021) demonstrated that a single bout of aerobic exercise provides an immediate and sustained (~47-min) benefit to task-switching performance as evidenced by a decreased magnitude unidirectional prosaccade switch-cost. In particular, Shukla and Heath (2021) had participants complete 20-min of heavy intensity (i.e., 80% of heart rate maximum) aerobic exercise via cycle ergometer and reported a 14 ms decrease in the unidirectional prosaccade switch-cost from a pre- to postexercise assessment and further observed that this benefit was sustained for up to 47-min postexercise (see also Heath and Shukla, 2020).

In the present work, participants (*N* = 40) completed baseline pro- and antisaccades arranged in an AABB task-switching schedule (i.e., A = prosaccade, B = antisaccade) and were then exposed to a validated acute stressor (i.e., Trier Social Stress Test: TSST). Subsequently, participants completed a second set of pro- and antisaccade task-switching trials after which they were assigned to an Exercise or Rest group. The Exercise group (*n* = 20) completed 20-min of the aforementioned exercise protocol, whereas the Rest group (*n* = 20) sat on the cycle ergometer without exercising. Following the Exercise and Rest intervention, participants completed a final set of pro- and antisaccade task-switching trials. Hence, the current protocol provides a basis to quantify whether a stress-induced impairment in task-switching is ameliorated via a 20-min bout of aerobic exercise. As for research predictions, it is anticipated that exposure to the TSST will result in a reliable increase in the magnitude of the unidirectional prosaccade switch-cost; that is, acute stress-induction is predicted to impair cognitive flexibility. In turn, it is anticipated that the unidirectional prosaccade switch-cost *magnitude* will return to baseline following the Exercise—but not Rest—intervention. In other words, we predict that the exercise intervention will attenuate the residual effects of stress induction on the cognitive flexibility component of executive function.

## Materials and methods

### Participants

Forty participants (21 female and 19 male, mean age = 22, SD = 3, age range = 19–30) volunteered from the Western University community. An *a priori* sample size was generated based on the effect size derived from an independent-samples *t*-test contrasting pre- and postexercise antisaccade RTs (α = 0.05, power = 0.95, dz. = 1.19; Petrella et al., 2019). All participants had normal or corrected-to-normal vision, self-declared being right-hand dominant, reported no history of neurological impairment (including concussion) or eye injury, and reported not taking medication for anxiety, depression, or a related mental health condition, nor did they take any medication that may impact their response to exercise. All participants attained a complete score on the Physical Activity Readiness Questionnaire for Everyone (PAR-Q+ 2021) and completed the Godin Leisure-Time Exercise Questionnaire (Godin, 2011) (Mean = 52, SD =24, Range = 28–99). All participants abstained from engaging in strenuous exercise, caffeine, and alcohol use 12 h prior to the experimental protocol. Participants were also instructed to obtain 8 h of sleep the night prior to the experimental protocol. All study procedures were conducted between 12:00 p.m. and 3:00 p.m. Participants provided informed written consent of a protocol approved by the Health Sciences Research Ethics Board (#118590), Western University. This study was conducted in line with the updated version of the Declaration of Helsinki except for participant registration in a public database.

### Procedure

### Stress induction

The Trier Social Stress Test (TSST) is an ecologically valid stressor that has been used in hundreds of studies to explore the impact of acute stress of psychological and physiological processes (Kirschbaum et al., 1993; Dickerson and Kemeny, 2004). For the TSST participants were informed that they would perform a filmed task where they would present a job interview-style speech to a panel of two judges trained in public speaking. Notably, participants were not familiar with the individuals who served on the mock judging panel. A camera was visible during the filmed speech to induce a state of social evaluation; however, the camera did not record participants’ performance, and following the completion of the study participants were debriefed that filming did not take place. The participant was then left alone in the examination room for 10-min for speech preparation before the judges returned. Participants then delivered their 5-min speech after which they were instructed to perform serial subtractions of 13 from 1,022 (i.e., 1,022 minus 13; 996 minus 13; etc.) in front of the same panel of judges. The serial subtractions lasted for 5-min or until subjects reached 0 (see Birkett, 2011 for further details on the TSST protocol). The nomenclatures TSST-Speech and TSST-Math are subsequently used below to describe the TSST speech and serial subtraction components, respectively. Prior to and immediately following the TSST, participants completed a measure of state anxiety. Throughout all data collection participants wore a heart rate monitor (Polar H10 Wearlink + Coded Transmitter, Polar Electro Inc., Lake Success, NY, United States).

### Exercise intervention

Participants (*n* = 20; 11 females, mean age: 20, range: 19–24) assigned to the Exercise Group sat on a cycle ergometer (Monark 818E Ergometer, Monark Exercise AB, Vansbro, Sweden) and completed a single bout of exercise that entailed: (1) a 2.5-min warm-up at an intensity less than 50% of their predicted maximum heart rate (i.e., HR_max_; 220- age), (2) a step-transition to a 20-min session of heavy intensity exercise (i.e., 80% of HR_max_), (3) a 2.5 min cool-down matching the intensity associated with the warm-up. The 80% of HR_max_ intensity was used based on previous work by our group demonstrating that a 20-min bout of exercise at the same intensity improves task-switching (Heath and Shukla, 2020; Shukla et al., 2020; Shukla and Heath, 2021). To maintain participants’ desired heart rate, the resistance on the cycle ergometer was adjusted as necessary. The postexercise oculomotor assessment (see below) was completed once heart rate returned to below 100 beats per minute (bpm).

### Rest intervention

Participants (*n* = 20; 10 females, mean age: 23, range: 18–30) sat on the same ergometer used by the Exercise Group. Here, participants sat for an equivalent time period (i.e., 25-min) as the exercise intervention without cycling and heart rate was measured as per the Exercise Group. Participants chatted with the experimenter during this time. The post-rest intervention oculomotor assessment was completed following the same timeline as per the Exercise Group.

### Stress measures

**Figure 1:**
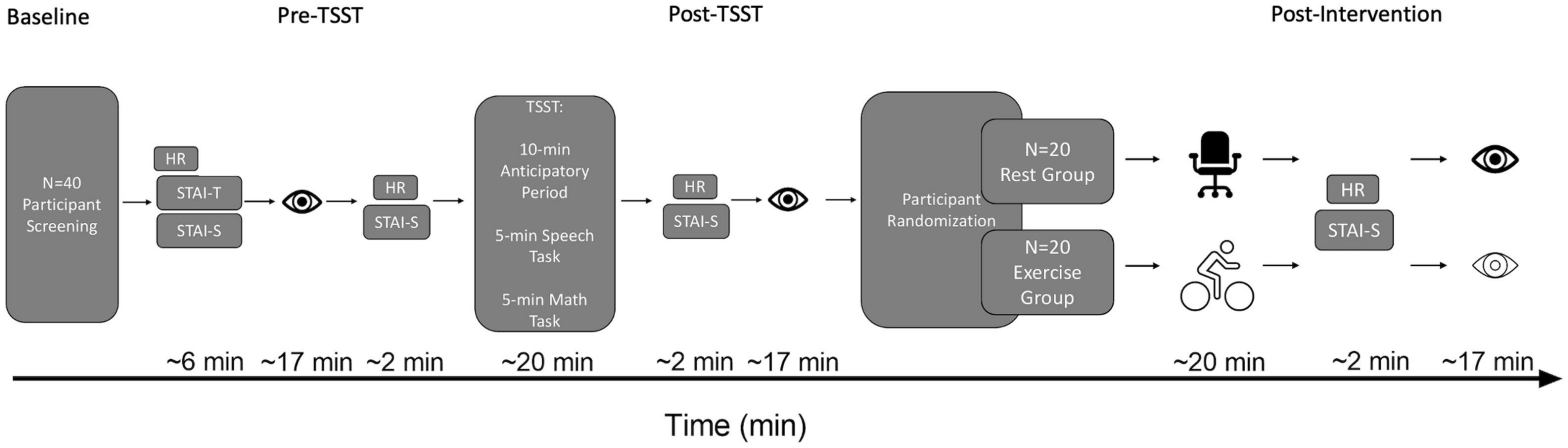
Schematic of the timeline of HR, STAI, and oculomotor assessments. The eye icon represents an oculomotor assessment. The desk chair icon represents the Rest group. The cyclist icon represents the Exercise group. HR, Heart rate; STAI-T, Trait Anxiety; STAI-S, State Anxiety; TSST, Trier Social Stress Test.

The State Trait Anxiety Inventory (STAI; Spielberger et al., 1983) was used to assess state and trait anxiety for all participants independent of group assignment. Twenty items assessed state anxiety and 20 items assessed trait anxiety with a higher score indicating increased anxiety. Trait anxiety was assessed upon entering the lab, whereas state anxiety was assessed at four time points: (1) upon entering the lab, (2) pre-TSST, (3) post-TSST, and (4) following either the Exercise or Rest intervention (i.e., post-intervention) (for timeline of events see Figure 1). Reliability estimates were computed via Cronbach’s alpha (α) and McDonald’s omega (ω)for the STAI State (Baseline) and Trait subscales. STAI State Baseline (α = 0.894, ω = 0.896) and Trait (α = 0.944, ω = 0.946).

### Oculomotor task

Prior to the TSST (i.e., pre-TSST), immediately following the TSST (i.e., post-TSST), and after Exercise and Rest interventions (i.e., post-intervention), participants completed an oculomotor task-switching paradigm (see Figure 1). For this assessment, participants sat on a height adjustable chair in front of a table (760 mm in height) with their head placed in a head/chin rest. Visual stimuli were presented on a 30-inch LCD monitor (60 Hz, 8 ms response rate, 1,280 × 960 pixels, Dell 3007WFP, Round Rock, TX, United States) placed 550 mm from the front edge of the tabletop and centered on participants’ midline and consisted of 1° luminance matched (40 cd/m^2^) red and green fixation crosses, as well as white target circles (2.5° diameter, 127 cd/m^2^) located 13° (i.e., proximal target) and 17° (i.e., distal target) to the left and right of fixation. The gaze location of participants’ left eye was measured via a video-based eye-tracking system (EyeLink 1000 Plus, SR Research, Ottawa, ON, Canada) sampling at 1,000 Hz. Prior to data collection a nine-point calibration of the viewing space was performed and confirmed via an immediate validation (<1° of error for each of the nine points in the calibration grid). Computer events were controlled via MATLAB (R2018b, The MathWorks, Natick, MA, United States) and the Psychophysics Toolbox extensions (v 3.0; Brainard, 1997; Kleiner et al., 2007) including the EyeLink Toolbox (Cornelissen et al., 2002). The lights in the experimental suite were extinguished during data collection.

**Figure 2:**
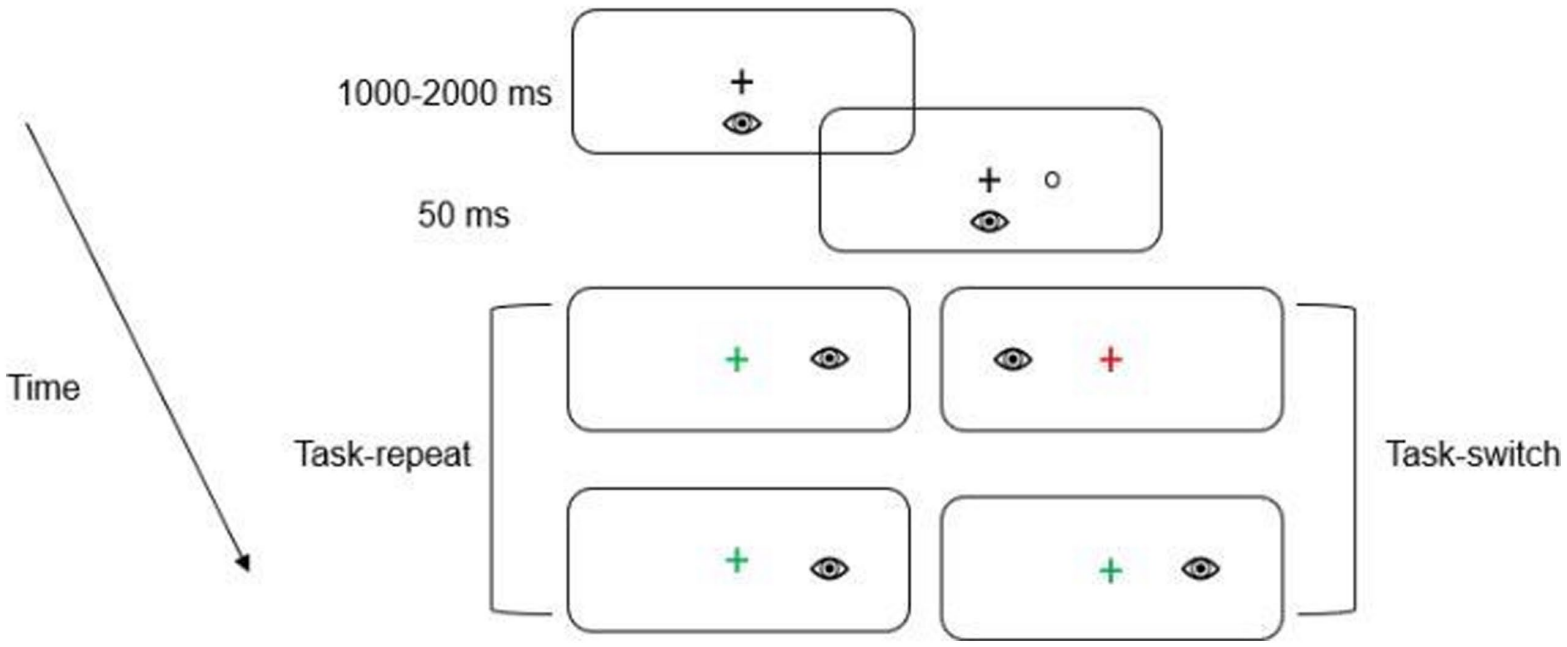
Schematic of the timeline of visual events. The first two panels depict the fixation cross and the presentation of the target, respectively. In this schematic, the green fixation cross denotes a saccade to the target’s veridical location (i.e., prosaccade), whereas the red fixation cross indicates a saccade to the target’s mirror-symmetrical (i.e., antisaccade) location. The subsequent panels depict an example of a task-repeat (i.e., prosaccade followed by a prosaccade) and task-switch (i.e., antisaccade followed by a prosaccade). Participants alternated between pro- and antisaccades after every second trial (i.e., AABB). For this schematic only a single target eccentricity presented in the right visual field is depicted.

A trial began with the presentation of a green or a red fixation cross which instructed participants to direct their gaze to its location. The color of the fixation cross indicated the nature of the upcoming trial. A green fixation cross indicated a prosaccade (i.e., saccade to veridical target location), whereas a red fixation cross indicated an antisaccade (i.e., saccade mirror-symmetrical to target location; see Figure 2).

Once a stable gaze was achieved (i.e., ±1.5° for 450 ms), a uniformly distributed randomized foreperiod between 1,000–2000 ms was initiated during which time the fixation cross remained visible. Following the foreperiod, a target was presented for 50 ms after which the target and fixation cross were extinguished (i.e., overlap paradigm). An overlap paradigm was used to minimize the frequency of directional errors (i.e., a prosaccade instead of an instructed antisaccade or vice versa) given that the primary metric for this study was RT for directionally correct responses. The onset of the target cued participants to pro- or antisaccade “as quickly and accurately as possible.” Pro- and antisaccades were arranged in a predictable AABB task-switching paradigm such that 40 prosaccade task-switch (i.e., prosaccade on trial N and an antisaccade on trial N-1) and 40 prosaccade task-repeat (i.e., prosaccade on trial N and N-1) trials were completed with an equivalent number of antisaccade task-switch and task-repeat trials (i.e., 160 trials). An AABB paradigm involves the conscious switching between distinct task sets (i.e., from a standard non-executive prosaccade task set to a non-standard, executive antisaccade task set and vice versa). Accordingly, the AABB task arrangement allows for the precise assessment of the cognitive flexibility domain of executive function (Diamond, 2013; see also above in more detail). For each oculomotor assessment an equal number of trials were pseudo-randomly presented to each target location (i.e., left proximal, left distal, right proximal, right distal). As shown in Figure 1, the oculomotor assessment of task-switching occurred pre-TSST, post-TSST and post-intervention. Each oculomotor assessment required ~17 min to complete (including calibration time) and is a time frame known to elicit a positive postexercise executive function benefit (Chang et al., 2012).

### Data reduction, dependent variables, and statistical analyses

#### Stress variables

Mean heart rate (HR) was calculated by averaging data pre-TSST, 5-min TSST (Speech), 5-min TSST (Math) and post-TSST. STAI scores were tabulated for trait and state subscales, and the latter were summed at baseline, pre-TSST, post-TSST, and post-intervention.

### Oculomotor variables

Point of gaze data were filtered offline using a dual-pass Butterworth filter employing a low-pass cut-off frequency of 15 Hz. A five-point central-finite difference algorithm was used to compute instantaneous velocities and accelerations. Saccade onset was determined via velocity and acceleration values that exceeded 30°/s and 8,000°/s^2^, respectively. Saccade offset was determined when velocity fell below 30°/s for 40 ms. Trials with missing data (e.g., blinks, signal loss) were excluded as were trials with: (1) an anticipatory response (RTs < 50 ms; Wenban-Smith and Findlay, 1991), (2) RTs > 2.5 standard deviations of a participant- and task-specific mean, and (3) trials with amplitudes <2° or >2.5 standard deviations of a participant- and task-specific mean (Gillen and Heath, 2014). Trials involving a directional error were excluded from subsequent analyses because such responses engage planning mechanisms distinct from their directionally correct counterparts (DeSimone et al., 2014). Less than 15% of trials were removed and only 2 and 4% of pro- and antisaccades entailed a directional error. The low error rate is attributed to the use of an overlap paradigm and the predictable nature of the AABB paradigm used here.

### Statistical analyses

Stress variables included HR and STAI scores. Mean HR values were analyzed via 4 (time: pre-TSST, concurrent TSST-Speech and TSST-Math, post-TSST) by 2 (group: Rest vs. Exercise) mixed-model ANOVA with group serving as the between-groups factor. STAI values were analyzed via 4 (time: baseline, pre-TSST, post-TSST, and post-intervention) by 2 (group: Rest vs. Exercise) with the former serving as the between-groups factor. This procedure is in keeping with previous studies examining HR and STAI scores during the TSST (Hellhammer and Schubert, 2012). Oculomotor dependent variables included RT (i.e., time from response cueing to saccade onset), saccade duration (i.e., time from saccade onset to saccade offset), and saccade gain variability (i.e., within-participant standard deviation of saccade amplitude/veridical target location). Oculomotor dependent variables were examined via 3 (time: pre-TSST, post-TSST, post-intervention) by 2 (task: prosaccade, antisaccade) by 2 (task transition: task-switch, task-repeat) by 2 group (i.e., Rest vs. Exercise) mixed-groups ANOVA. Huynh-Feldt corrections for violations for sphericity are reported where appropriate (corrected degrees of freedom reported to one decimal place) and an alpha level of 0.05 was used for all ANOVA models. Interactions and appropriate main effects were decomposed via simple effects (i.e., paired-samples *t*-tests). Where appropriate, Bayesian statistics were employed to confirm null results and the two one-sided test (TOST) statistic is reported to determine whether results were within an equivalence boundary (Lakens, 2017). The effect size used to compute the TOST statistic (*d*_z_ = 0.54) was derived from paired-samples *t*-tests comparing prosaccade task-switch and task-repeat trials following Exercise and Rest interventions.

## Results

### Stress measures

#### Heart rate

**Figure 3:**
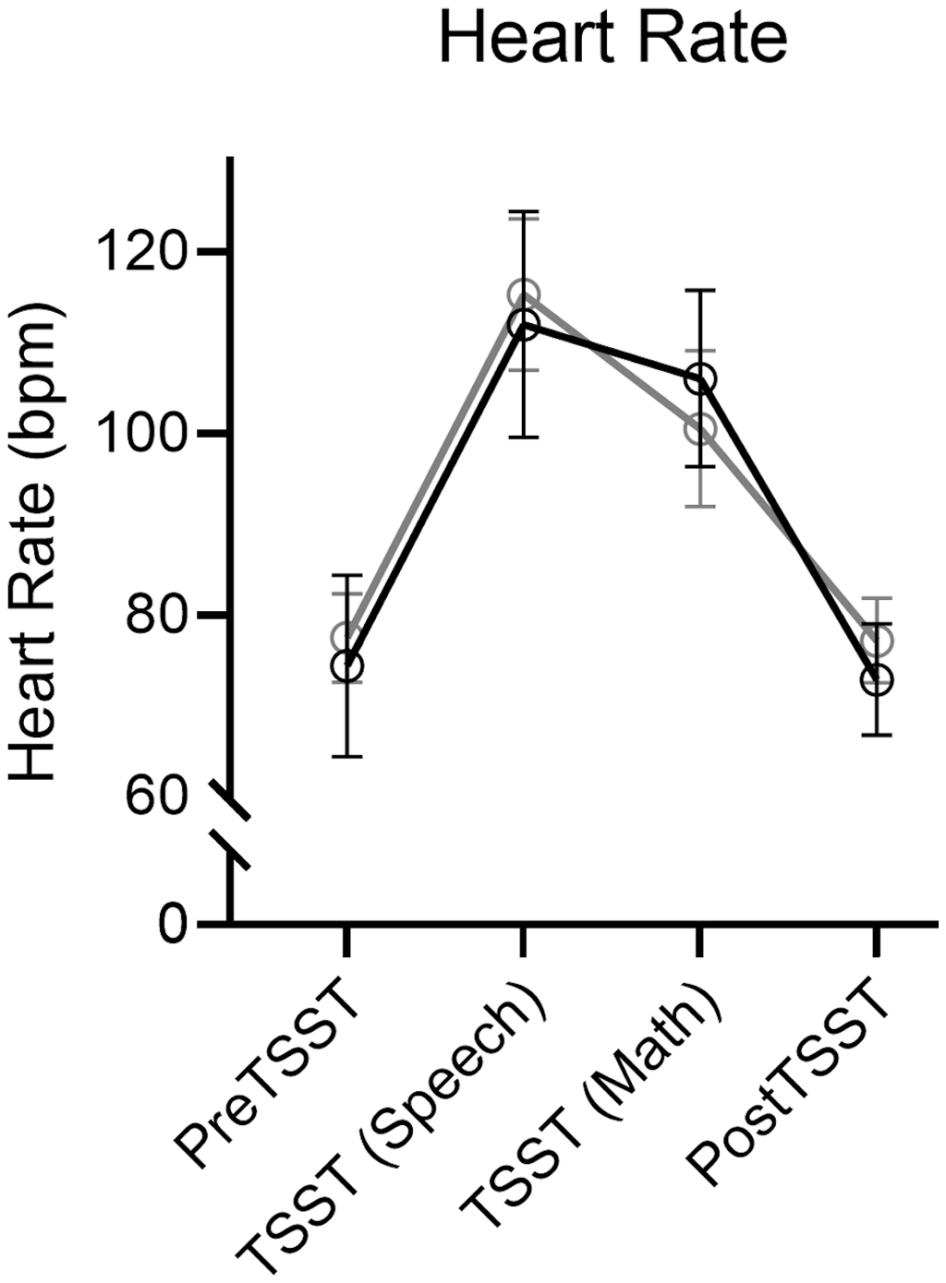
Mean heart rate (in beats/min: bpm) and associated 95% confidence intervals for Exercise and Rest groups at baseline, TSST-Speech, TSST-Math, and Post-TSST.

Results produced a main effect of time, *F*(3, 114) = 96.67, *p* < 0.001, η_p_^2^ = 0.72. Figure 3 shows an increase in HR during the TSST-Speech and TSST-Math portions in relation to the pre-TSST [all *t*(39) = −11.25, and − 9.28, *p*s < 0.001, *d*_z_ = −1.78 and − 1.47] and that values for the 10-min post-TSST did not reliably differ from the pre-TSST assessment [*t*(39) = 0.42, *p* = 0.67, *d*_z_ = 0.07]. Notably, a null time by group interaction, *F*(3, 114) = 1.32, *p* = 0.27, η_p_^2^ = 0.03, indicated that HR values did not reliably differ between Exercise and Rest groups.

#### State trait anxiety inventory

**Figure 4:**
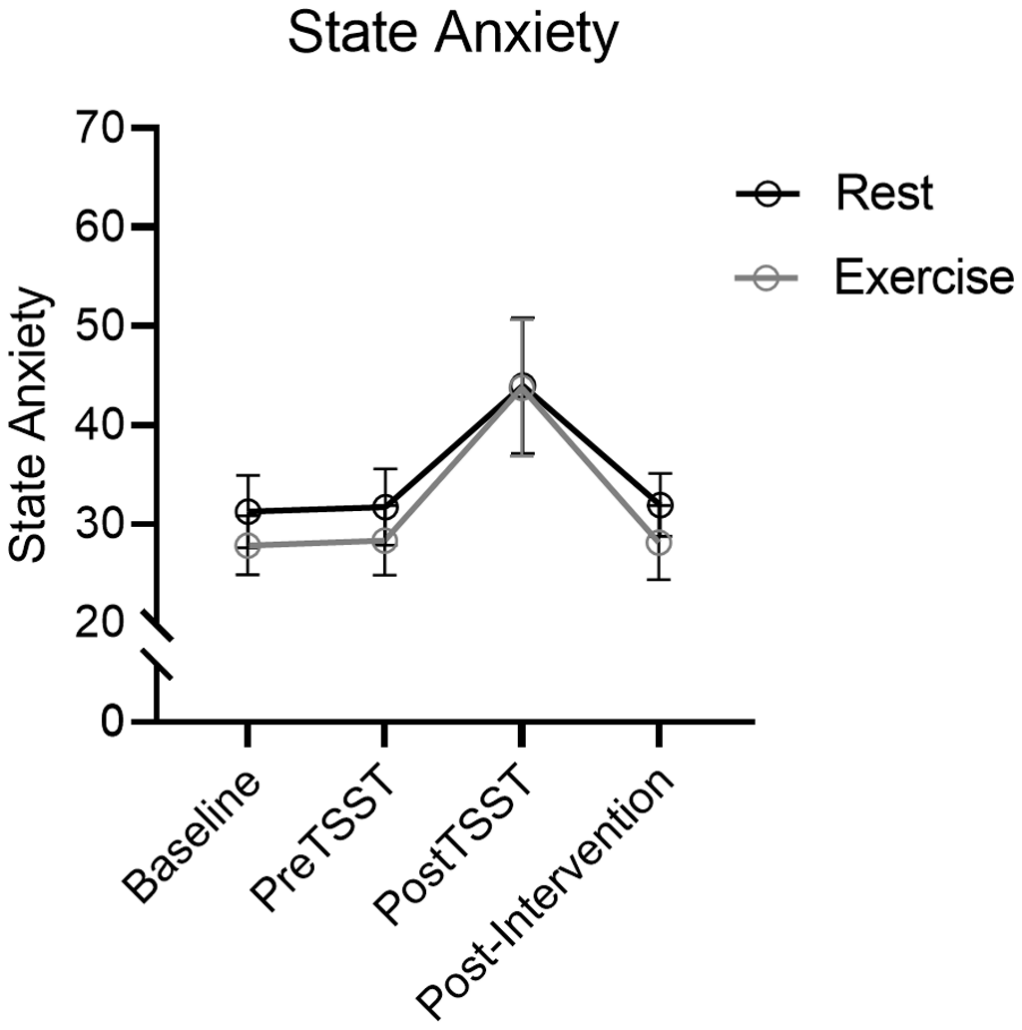
STAI mean values for Exercise and Rest groups at baseline, TSST-Speech, TSST-Math and Post-TSST. Error bars represent 95% within-participant confidence intervals.

Results produced a main effect of time, *F*(2.2, 83.9) = 108.03, *p* < 0.001, η_p_^2^ = 0.74. STAI scores increased from pre-TSST to post-TSST [*t*(39) = 6.12, *p* = <0.001, *d*_z_ = 0.97] (see Figure 4) however, a null time by group interaction, *F*(2.2, 83.9) = 1.03, *p* = 0.37, η_p_^2^ = 0.03, indicated that STAI scores did not reliably differ between Exercise and Rest groups.

### Oculomotor measures

#### Reaction time

**Figure 5:**
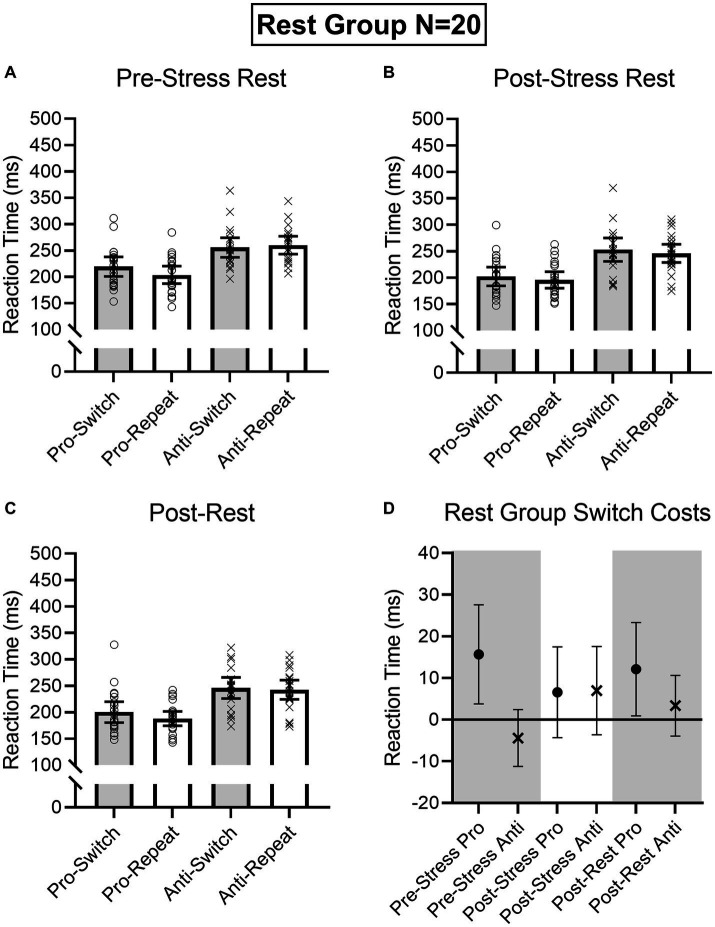
Rest group participant-specific pro- and antisaccade task-switch and task-repeat reaction times (i.e., open symbols) and associated group means at baseline **(A)**, post-TSST **(B)**, and post-Rest **(C)**. Error bars represent 95% within-participant confidence intervals. As well, the lower right panel **(D)** shows difference scores and 95% between-participant confidence intervals relating baseline reaction times to each subsequent oculomotor assessment. For this panel, the absence of overlap between the error bar and zero (i.e., the horizontal line) represents a reliable effect inclusive to a test of the null hypothesis.

**Figure 6:**
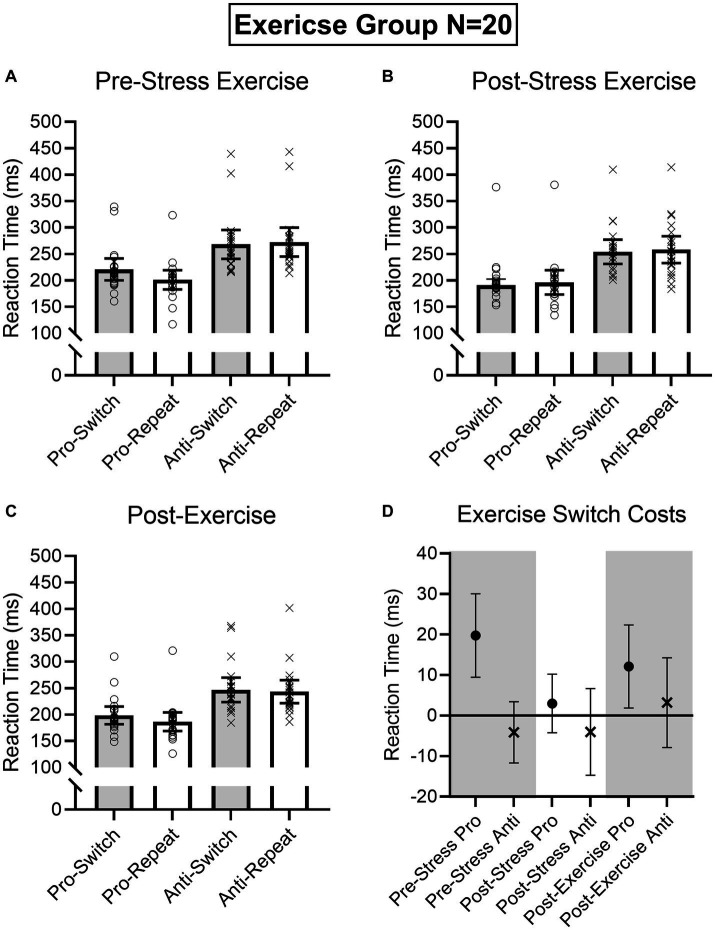
Exercise group participant-specific pro- and antisaccade task-switch and task-repeat reaction times (i.e., open symbols) and associated group means at baseline **(A)**, post-TSST **(B)**, and post-Exercise **(C)**. Error bars represent 95% within-participant confidence intervals. As well, the lower right panel **(D)** shows difference scores and 95% between-participant confidence intervals relating baseline reaction times to each subsequent oculomotor assessment. For this panel, the absence of overlap between the error bar and zero (i.e., the horizontal line) represents a reliable effect inclusive to a test of the null hypothesis.

Results produced main effects of time, *F*(2, 76) = 16.91, *p* < 0.001, η_p_^2^ = 0.31, task, *F*(1, 38) = 162.24, *p* < 0.001, η_p_^2^ = 0.81, and task transition, *F*(1, 38) = 8.48, *p* = 0.006, η_p_^2^ = 0.18, as well as interactions involving task by task transition, *F*(1, 38) = 12.54, *p* = 0.001, η_p_^2^ = 0.25, and time by task by task transition, *F*(2, 76) = 9.69, *p* < 0.001, η_p_^2^ = 0.20. As expected, RTs for prosaccades (201 ms, SD = 36) were shorter than antisaccades (254 ms, SD = 43)—a finding consistent across task-switch and task-repeat trials. In decomposing the three-way interaction, we computed task by task transition ANOVAs separately for the Pre-TSST, Post-TSST, and Post-intervention oculomotor assessments. Figures 5, 6 demonstrate that pre-TSST and post-intervention assessments produced task by task transition interactions, all *F*(1, 38) = 27.22 and 7.70, *p*s < 0.001 and 0.009, all η_p_^2^ = 0.42 and 0.17, indicating that prosaccade task-switch RTs were longer than their task-repeat counterparts [all *t*(39) = 4.76 and 3.39 for pre-TSST and post-intervention, respectively, *p*s < 0.001 and = 0.02, *d*_z_ = 0.75 and 0.54], whereas antisaccade task-switch and task-repeat trials did not reliably differ [all *t*(39) < −1.78, *p*s > 0.08, *d*_z_ < −0.28]. In turn, the post-TSST assessment did not yield a task by task-transition interaction, *F*(1, 38) = 0.49, *p* = 0.49, all η_p_^2^ = 0.01. In other words, the unidirectional prosaccade switch-cost was observed pre-TSST and following Exercise and Rest interventions but not following TSST administration. Further, and given the nature of our research question, we note that results did not yield a main effect of group, *F*(1, 38) = 0.05, *p* = 0.82, η_p_^2^ = 0.001, nor any higher-order interaction involving group, all *F*s(2, 76) < 1.47, *p*s > 0.24, all η_p_^2^ < 0.04. As well, we note that TOST statistics indicated that post-TSST switch-costs for the Exercise (12 ms, SD = 24) and Rest (12 ms, SD = 22) groups were within an equivalence boundary, *t*(37.7) = 1.71, *p* = 0.048 (*d*_z_ = 0.54).

Given the importance of evaluating the null findings, in addition to the frequentist statistics provided above, we conducted single-sample Bayesian statistics comparing prosaccade switch-costs (i.e., task-switch minus task-repeat trial) separately for each assessment interval (i.e., pre-TSST, post-TSS and post-intervention). Bayes factors for the alternative hypotheses (i.e., BF10) at pre-TSST, post-TSST and post-intervention were 803.36, 0.507, and 19.94, respectively. In line with van Doorn et al.’s (2021) nomenclature such findings evidence “very strong” and “strong” evidence for the alternative hypothesis, respectively, for the pre-TSST and post-intervention assessments. In turn, the value for the post-TSST provided less than ‘anecdotal’ evidence for the alternative hypothesis. Moreover, evaluation of the post-TSST Bayes factor for the null hypothesis (i.e., BF01 = 4.24) indicated that the null hypothesis was more likely than the alternative. In other words, our exercise manipulation did not modulate the unidirectional prosaccade switch-cost.

#### Saccade duration and gain variability

Results yielded main effects of task, all *F*s(1, 38) = 20.91 and 84.36, *p*s < 0.001, all η_p_^2^ = 0.36 and 0.69. Antisaccade durations were longer (75 ms, SD = 14) and endpoints were more variable (0.22, SD = 0.08) than prosaccades (saccade duration: 68 ms, SD = 8; saccade gain: 0.12, SD = 0.08).

## Discussion

We examined the effects of acute stress and a single bout of aerobic exercise on cognitive flexibility via the AABB pro- and antisaccade task-switching paradigm. In addressing our findings, commentary is first provided regarding the effects of acute stress on cognitive flexibility before discussing whether our exercise manipulation modified a stress-induced disruption to cognitive flexibility.

### The TSST produces a reliable and transient stress response

HR was elevated from pre-TSST to TSST-Speech (38 bpm) and TSST-Math (27 bpm) and are values on par to the respective 31 bpm and 27 bpm HR increases in TSST-Speech and TSST-Math reported by Rosenbaum et al. (2018). HR values returned to pre-TSST values approximately 15 min after the TSST. In terms of STAI results, values increased from pre- to post-TSST and the magnitude of this increase is on par with previous work (e.g., Birkett, 2011). Hence, the HR and STAI data demonstrate the TSST elicited a reliable stress response and thus serves as a framework by which to assess stress-induced changes in cognitive flexibility.

### An acute stressor boosts task- switching performance

Pre-TSST prosaccade task-switch RTs were longer than their task-repeat counterparts, whereas antisaccade task-switch and task-repeat RTs did not differ. In other words, a unidirectional prosaccade switch-cost was present pre-stress and is in line with prior work by our group (Weiler and Heath, 2012a, 2014a; Weiler et al., 2015; Heath et al., 2016; Tari and Heath, 2019 Tari et al., 2019 Heath and Shukla, 2020; Shukla et al., 2020; Shukla and Heath, 2021) and others (Manoach et al., 2007; Chan and DeSouza, 2013). When considering this finding, it is important to note that some have argued that the RT difference between prosaccade task-switch and task-repeat trials may reflect a repetition; that is, the second of two consecutively performed prosaccades is associated with an RT reduction. Notably, however, a purpose-designed study by our group showed that prosaccade task-repetition trials yield RTs on par to prosaccades executed in a separate block of trials (Weiler and Heath, 2014a). In other words, results support the assertion that the RT difference between prosaccade task-switch and task-repeat trials reflect a switch-cost wherein the planning times for the former trial-type are delayed due to a task-set inertia from a previously completed antisaccade trial (Weiler and Heath, 2014a; Weiler et al., 2015; see also Tari et al., 2019).

Although a unidirectional prosaccade switch-cost was observed pre-stress, it was not present post-stress. The absence of a switch-cost post-stress cannot be attributed to an “implicit – or explicit – control strategy” (Heath and Shukla, 2020) designed to increase prosaccade task-switch trial planning times (i.e., speed-accuracy trade-off) given that saccade durations and endpoint accuracy for all trial-types did not vary across the different assessment intervals. Moreover, and as will be discussed in more detail below, the absence of a prosaccade switch-cost cannot be attributed to a practice-effect given that a switch-cost was observed at the Exercise and Rest group post-intervention assessments. Indeed, at the outset we were surprised by the absence of a post-TSST switch-cost given that a meta-analysis of the extant (Shields et al., 2016) concluded that cognitive flexibility is adversely impacted by an acute stressor. However, it is important to acknowledge that prior work has reported findings similar to those observed here. For example, Gabrys et al. (2019) investigated the effects of the TSST on cognitive flexibility via the computer-based Berg Card Sorting Task (BCST). The BCST comprises of an electronic card deck wherein each card contains a different assortment of one of four colors, shapes, and quantities. Target cards are presented and the participants is required to sort response cards according to different sorting rules (which are unknown to the participant). The primary outcome for the BCST was perseverative errors (i.e., sorting cards in accordance with the previous sorting rule) and results showed that participants exposed to the TSST exhibited reduced perseverative errors compared to a control group. Similarly, Goldfarb et al. (2017) examined the effects of the cold pressor task on cognitive flexibility via the modified delayed match-to-sample task. In the first phase of the delayed match-to-sample task, participants learned two colored stimuli, whereas in the second phase participants were required to either remember the colored stimuli or to forget the stimuli and learn two novel stimuli (i.e., a task requiring cognitive flexibility). Participants exposed to the cold pressor task demonstrated improved cognitive flexibility which the authors asserted may be due to the fact that the ability to incorporate newly relevant information relies on the dorsal striatum, which has been documented to show enhanced functioning following acute stress (Packard, 2009; Delgadoy Palacios et al., 2014). Hence, there is evidence indicating that a stressor may serve to focus or limit attentional resources and support disengagement from task-irrelevant information. In other words, an acute stressor may provide a level of task-engagement and attentional selectively supporting a transient benefit to task-switching.

### Exercise does not modulate task-switching following stress

A single bout of aerobic exercise did not modulate post-stress task-switching performance. Instead, our post-stress assessment revealed an equivalent magnitude unidirectional prosaccade switch-cost for Exercise and Rest groups. This represents an unexpected result given a myriad of previous studies showing that a single bout of aerobic exercise provides a positive benefit to each core component of executive function (for reviews see, Lambourne and Tomporowski, 2010; Chang et al., 2012; Morris et al., 2020). Indeed, a previous investigation in our lab showed a 14 ms decrease (*d*_z_ = 0.72) to the magnitude of the unidirectional switch-cost when completed immediately and up to 47-min following a 20-min single bout of heavy intensity aerobic exercise (i.e., the same exercise protocol as used here; see Heath and Shukla, 2020; Shukla et al., 2020; Shukla and Heath, 2021). In contextualizing the present work with earlier work, the prior studies did not employ an acute stress manipulation. Thus, in the subsequent section we address why an acute stressor may “blunt” a postexercise benefit to executive function.

### Neurobiological mechanisms

The interaction of several neurobiological systems likely influences the relationship between stress, exercise and cognitive flexibility (for reviews of stress on neural systems and the prefrontal cortex see, Arnsten, 2009; Joëls and Baram, 2009). It is well-documented that immediately after a stressor there is a rapid increase in the concentration of monoamines and neuropeptides (e.g., dopamine, serotonin, norepinephrine, and corticotropin-releasing hormone) in frontoparietal regions (Joëls and Baram, 2009), and that such a change may alter executive function. For example, increases in dopamine and norepinephrine alter neuronal firing patterns in the prefrontal cortex (Joëls et al., 2006; McMorris, 2016) and this state change in animal models has been shown to facilitate high-level movement planning and decision-making (Barbas, 2000) and in humans has been shown to elicit improved executive function via increased task-based attentional focus (McMorris, 2016). A second mechanism may reflect the release of cortisol from the HPA axis (Joëls and Baram, 2009). Cortisol exerts both fast-acting (i.e., non-genomic) and slow-acting (i.e., genomic) effects on neurobiological systems (Joëls et al., 2011) and is documented to affect the activity of local neural networks supporting executive function (Reul and Kloet, 1985). For example, Schwabe et al. (2013) administered to participants a mineralocorticoid antagonist (i.e., an agent that blocks the stimulating effects of cortisol) or a placebo prior to a stressor (i.e., cold pressor with element of social evaluation) or a non-stressor. After the stressor, participants performed the stop-signal task (i.e., task of inhibitory control) and results indicated that exposure to a stressor enhanced inhibitory control; however, this result was not present following when the stressor was preceded by a mineralocorticoid antagonism. Put another way, acute stress may modulate executive function, in part, via cortisol.

In addressing the absence of a postexercise benefit to task-switching, we note the time-dependent release of cortisol exerts fast (i.e., non-genomic) and slow (i.e., genomic) effects. As the final oculomotor assessment was administered approximately 55 min following completion of the stress-inducing event (i.e., the TSST), this likely coincided with the beginning of the “slow, genomic effects” of cortisol (Joëls and Baram, 2009). The genomic effects of cortisol have been documented to impede components of executive function (Shields et al., 2015) and may blunt the putative neuroprotective benefit that a single bout of exercise provides to task-switching.

### Study limitations

First, cortisol was not measured and therefore precludes direct interpretation of how it may have influenced task-switching efficiency. Second, the present work employed only a 20-min bout of high-intensity aerobic exercise and evaluated cognitive flexibility via task-switching at a single postexercise timepoint (i.e., immediately after exercise). Thus, it is unclear whether different exercise durations across the continuum of exercise intensities would induce a similar result and it is unclear whether exercise may produce a delayed improvement in executive function. Third, our results are limited to healthy and recreationally active young adults. As the current work does not include a pre-registered replication, we would strongly encourage future works to consider a replication of this study in similarly aged, middle-aged, and old-aged healthy, cognitively intact individuals. Indeed, given that older adults show differing levels of reactivity to stress-inducing events (Uchino et al., 2005) and to exercise (Ludyga et al., 2016) it remains unknown whether our findings extend to broader populations. Last, including another measure of arousal such as pupillometry (see Ayala and Heath, 2021) would afford insight into the mechanisms underlying changes to cognitive flexibility as a result of acute stress.

## Conclusion

The current findings provide insight into the complex dynamics between acute stress and cognitive flexibility. Namely, acute stress reduces the magnitude of a unidirectional prosaccade switch-cost and that a single bout of heavy intensity exercise did not differentially modulate these effects compared to rest.

## Data availability statement

The raw data supporting the conclusions of this article will be made available by the authors, without undue reservation.

## Ethics statement

The studies involving human participants were reviewed and approved by Western University Research Ethics Board. The patients/participants provided their written informed consent to participate in this study.

## Author contributions

AM, BT, HP, and MH conceptualized the study, contributed to the methodology, and data analysis. AM, JA, and MS collected the data. AM wrote the initial manuscript draft. HP and MH reviewed and edited the manuscript, supervised the study. MH acquired grant funding. All authors contributed to the article and approved the submitted version.

## Funding

This work was supported by the Natural Sciences and Engineering Research Council Discovery Grant (MH).

## Conflict of interest

The authors declare that the research was conducted in the absence of any commercial or financial relationships that could be construed as a potential conflict of interest.

## Publisher’s note

All claims expressed in this article are solely those of the authors and do not necessarily represent those of their affiliated organizations, or those of the publisher, the editors and the reviewers. Any product that may be evaluated in this article, or claim that may be made by its manufacturer, is not guaranteed or endorsed by the publisher.
